# Classification of QRS complexes to detect Premature Ventricular Contraction using machine learning techniques

**DOI:** 10.1371/journal.pone.0268555

**Published:** 2022-08-18

**Authors:** Fabiola De Marco, Filomena Ferrucci, Michele Risi, Genoveffa Tortora

**Affiliations:** Department of Computer Science, University of Salerno, Fisciano, Italy; University of Minnesota, UNITED STATES

## Abstract

Detection of Premature Ventricular Contractions (PVC) is of crucial importance in the cardiology field, not only to improve the health system but also to reduce the workload of experts who analyze electrocardiograms (ECG) manually. PVC is a non-harmful common occurrence represented by extra heartbeats, whose diagnosis is not always easily identifiable, especially when done by long-term manual ECG analysis. In some cases, it may lead to disastrous consequences when associated with other pathologies. This work introduces an approach to identify PVCs using machine learning techniques without feature extraction and cross-validation techniques. In particular, a group of six classifiers has been used: Decision Tree, Random Forest, Long-Short Term Memory (LSTM), Bidirectional LSTM, ResNet-18, MobileNetv2, and ShuffleNet. Two types of experiments have been performed on data extracted from the MIT-BIH Arrhythmia database: *(i)* the original dataset and *(ii)* the balanced dataset. MobileNetv2 came in first in both experiments with high performance and promising results for PVCs’ final diagnosis. The final results showed 99.90% of accuracy in the first experiment and 99.00% in the second one, despite no feature detection techniques were used. The approach we used, which was focused on classification without using feature extraction and cross-validation techniques, allowed us to provide excellent performance and obtain better results. Finally, this research defines as first step toward understanding the explanations for deep learning models’ incorrect classifications.

## Introduction

The cardiovascular disease (CVD) is still the leading cause of death worldwide. According to the World Health Organization (WHO), in 2017 more than 17.9 million people died from cardiovascular disease (31% of all deaths worldwide), and over three-quarters of CVD deaths occurred in low-cost countries and middle-income countries [1]. Around 400,000 deaths from cardiac arrest are reported annually in Europe: about 1,095 deaths every day, about 45 per hour. A lifestyle marked by smoking, alcohol, and substance abuse, but also a sedentary lifestyle and a careless diet can promote the onset of cardiovascular disease. Risk assessment and prevention through appropriate diagnosis and effective treatment is one of the main goals of the scientific community to predict and prevent future cardiovascular disease mortality [2]. CVDs are therefore multifactorial, i.e., associated with multiple risk factors, and can be defined as all pathologies affecting the heart and blood vessels, such as various forms of arrhythmia, heart valve pathologies, heart failure, and so on.

**Fig 1:**
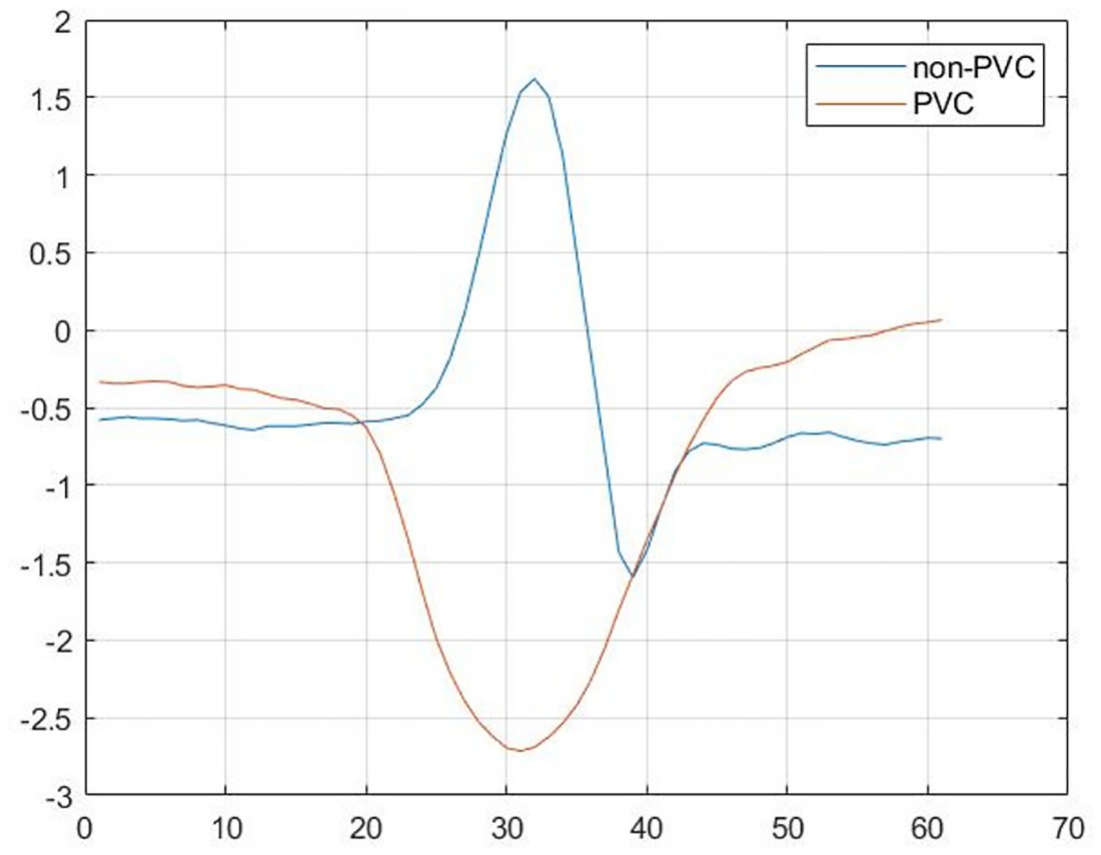
The difference between two signals: Normal and PVC (abnormal).

Premature Ventricular Contraction (PVC) is an additional heartbeat that occurs in one of the two heart ventricles that delays the normal pumping order, first the atria, then the ventricles. Typically, these extra contractions beats are faster than the next expected normal heartbeat, causing the chest to feel a fluttering or a skipped beat. The causes aren’t always evident. The cells of the ventricles can become electrically unstable due to a variety of factors, including heart disease or scarring, as well as the abuse of certain medications (decongestants and antihistamines), drugs, or alcohol, or an increase in adrenaline levels in the body. Occasional PVCs do not need treatment because many people have a similar event and it is not dangerous, but repeated PVCs or certain habits can increase the risk of having problems with heart rhythm (arrhythmias) or weakening of the heart muscle (cardiomyopathy). Rarely, when accompanied by heart failure, repeated PVCs may result in chaotic, risky heart rhythms and likely sudden cardiac death. Historically, the diagnosis of PVC is made by experts who evaluate and identify the electrocardiogram’s (ECG) characteristic parameters, but this method is too sluggish and inefficient. Therefore, early identification and diagnosis of PVCs are necessary to prevent not only the above-mentioned complications but also to alleviate the experts’ workloads. The ECG is a diagnostic test that gives a great deal of knowledge about the rhythm of the heart and the existence of abnormal waves, as depicted in Fig 1. The heartbeat of a healthy person has three characteristics: the P wave, the Q, R, and S waves which make up the QRS complex, and the T wave [3].

A great effort has been done to automate the ECG signal analysis process and to design a classifier that discriminated against healthy signals from those indicative of arrhythmia. However, the heart is a complex organ, and many new and different types of arrhythmias may occur over time.

The main challenges in the classification of arrhythmias through the ECG can be divided into two parts: the extraction of features and the choice of classification algorithms [4].

Traditional machine learning techniques have initially been widely used in the literature for the analysis of different arrhythmias [4] and then deep learning techniques have been explored, resulting in major cardiology outcomes. Decision Trees are classifiers commonly used, either alone or associated with other models such as K-Nearest Neighbors (KNN), and Support Vector Machine (SVM) [5]. Kaya and Pehlivan developed an effective and comparative approach for the classification of arrhythmias intending to improve the classification performance of PVCs [6]. They examined performance over time series but with reduced characteristics equivalent to that of the ECG signals. Often the data is pre-processed for the detection of the QRS complex using the Pan Tompkins algorithm [6] for the automatic classification of different types of beats or the Pan Tompkins algorithm together with the Windowing and thresholding method [7]. In the case of Kaya and Pehlivan the extraction is performed to classify three different types of beats: normal sinus rhythm (N), PVC, and left bundle branch block (LBBB). In [7], twenty-five features are extracted for each beat and a completely new method is introduced. In the case of Geeta and Naveen the Decision Tree (DT) are used to classify the PVCs. Ensemble learning techniques, such as Random Forest, are also used to combine three specific characteristics: RR intervals, QRS area, and R peak amplitude [8].

However, the use of Deep Learning has grown exponentially in recent years, as it has proved to be one of the most accurate and effective techniques in a wide range of medical problems, such as diagnosis, prediction, and intervention. Several Convolutional Neural Network structures have been used, as in [9], where the kernel size of the starting level is extended to classify ECG beats as normal sinus rhythm (N), PVC, premature atrial contraction (APC), right bundle arrhythmia (RBBB) and LBBB. However, one of the most common models is the Long-Short Term Memory (LSTM), a particular Recurrent Neural Network, which detects and partially resolves feature extraction problems [10]. All of the methods mentioned above, including [7, 8] have limitations, such as the extensive uses of pre-processing techniques and cardiologists have difficulties using them since the results are not always immediate and have a high proportion of error rate. Even though feature extraction techniques are not used in many cases, the aforementioned deep learning techniques, such as [9, 10] only partially solve these limitations due to the use of cross-validation techniques for training the models.

The introduction of innovative technologies made the use of feature extraction and cross-validation techniques unnecessary, significantly improving performance. In this scenario, the goal of our work was to use specific and appropriate machine learning techniques, in particular, a group of six classifiers such as Decision Tree (DT), Random Forest (RF), ResNet-18, MobileNetv2, ShuffleNet, Long-Short Term Memory (LSMT), and Bidirectional LSTM (BLSTM), which enabled the avoidance of feature extraction and cross-validation techniques achieving excellent and greater performance than that reported by the state of the art.

The dataset utilized was extrapolated from the MIT-BIH Arrhythmia Database, on which two types of tests were done to obtain a better performance analysis: *(i)* with the original dataset, *(ii)* with the balanced dataset.

MobileNetV2 was the network that performed the best in both experiments among the strategies utilized in this study and in comparison to the methodologies proposed in the literature. The obtained results demonstrate excellent performance without the use of pre-processing techniques and highlight how the structure and size of the dataset have the greatest influence on the final outcomes. Finally, this approach represents a significant contribution to the investigation of the models’ misclassification reasons.

The rest of the paper is organized as follows. Section *Methods* describes the chosen dataset and the classifiers used in both experiments. Section *Results and Discussion* presents the experiment’s performance and relatives consideration. Finally, *Conclusion* concludes the paper.

## Methods

This section describes the dataset and the methodology used to detect and classify the non-PVC QRS complexes from the PVC QRS complexes for the various experiments.

### Dataset

The dataset used in this research, the MIT-BIH Arrhythmia Database (MIT-BIH), was the first universally available set consisting of standard tests for the evaluation of arrhythmia detectors. The basic technique used for monitoring transient aspects of cardiac electrical activity is the long-term ECG, inexpensive and non-invasive, usually with a length of 24 hours [11]. The dataset was developed by the Research Team of the Beth Israel Hospital Arrhythmia Laboratory (BIH), now Beth Israel Deaconess Medical Center, who recorded, digitized, and annotated several patients’ long-term ECG recordings. In order to promote the work in the field of Cardiology and to facilitate rigorously reproducible and scientifically comparable tests of the various algorithms developed, these recordings were available to the research community from the outset. MIT-BIH consists of 48 half-hour extracts from each two-channel ECG recording, 24 hours a day, obtained from 47 subjects. Of these, 23 subjects form the *“100”* series and have been randomly selected from a collection of over 4000 Holter recordings, while the remaining 25 subjects that are the *“200”* series have been specifically selected to include examples of very rare arrhythmias in the database but clinically significant that could not be well represented by purely random samples. Subjects included 25 men between the ages of 32 and 89 and 22 women between the ages of 23 and 89; nearly 60% of subjects were hospitalized and the remaining 40% were outpatients. As expected in clinical practice, ECG recordings differed between subjects, as surgical medications and anatomical differences did not allow the use in all cases of the same electrode placement. The recordings were digitized at 360 samples per second per channel with a resolution of 11 bits over a range of 10 mV.

The dataset used in this paper, obtained from the above mentioned database, contains a total of 82,178 items from 47 patients. Each ECG record has been extrapolated using a sampling method based on 169 ms time intervals that completely covered the QRS complex curve. The original dataset contains precisely 75,048 elements for the non-PVC QRS complexes and 7,130 elements for the PVC QRS complexes. For this reason, there is a first phase in which the dataset is balanced using the subsampling technique for reducing data size by picking a subset of the original data; two types of experiments are carried out: *(i)* with the original dataset, *(ii)* with the balanced dataset. In the second phase the raw data have been converted into color images in order to correctly train the CNNs. Each image represents an entire sample (row) from the original dataset. As a result, two types of dataset have been obtained: the original dataset composed of raw data and the dataset made up of images. In both experiments, the dataset was divided for CNNs into 70% for training, 15% for validation, and 15% for testing. For the other models, however, the dataset was divided into 80% for training and 20% for validation.

The percentage of presence of non-PVC and PVC QRS complexes in the first experiment was 91% and 8%, respectively, with the training set consisting of 60038 non-PVC samples and 5704 PVC samples, and the test set consisting of 15010 samples of non-PVC QRS complexes and 1426 PVC QRS complexes. Instead, in the second experiment (with the balanced dataset), the percentage of the existence of non-PVC and PVC QRS complexes is exactly 50% in both the training and test sets, i.e. 5704 non-PVC and 5704 PVC examples in the training set and 1426 non-PVC and 1426 PVC examples in the test set.

### QRS complexes classification

This subsection describes the chosen machine learning techniques for the classification of QRS complexes.

**Fig 2:**
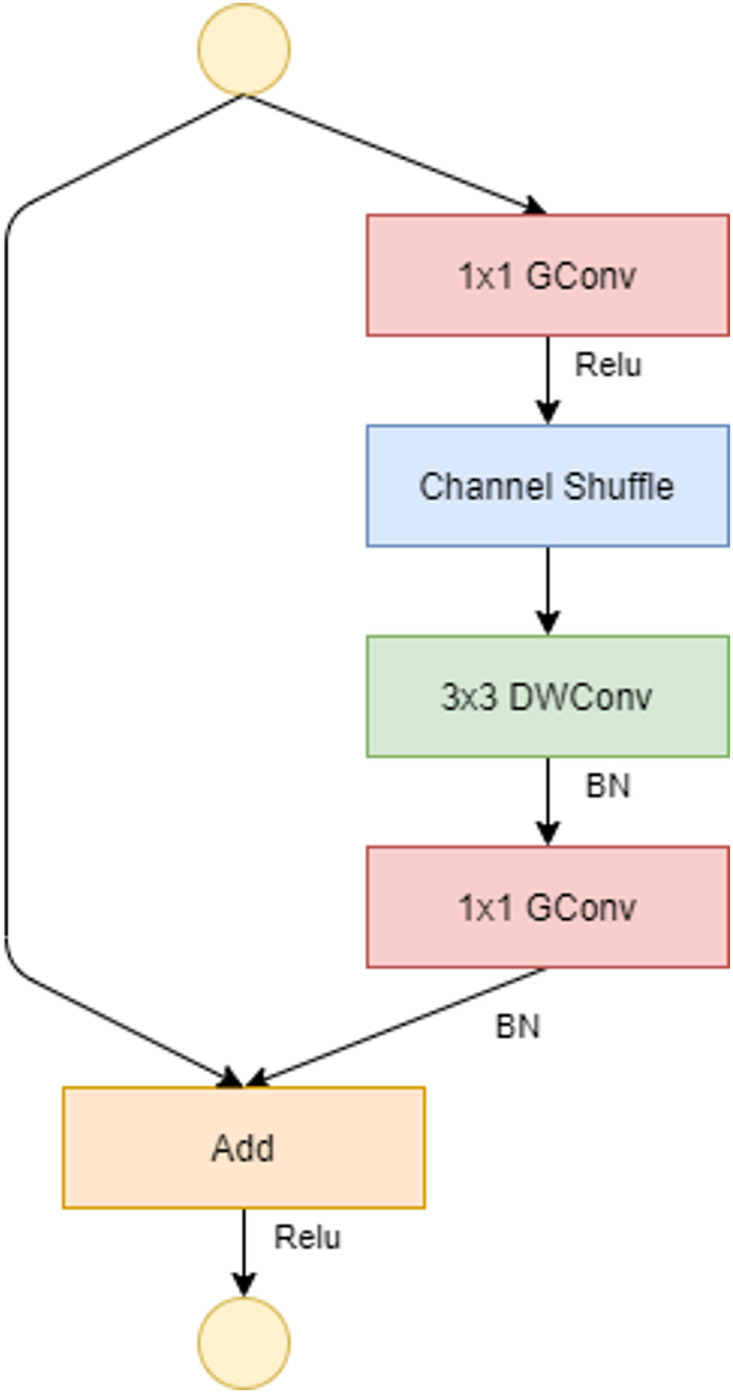
Methodology to classify QRS complexes.

The dataset obtained was used to train the models and the performance was compared in order to evaluate the best model in both experiments. The proposed approach is detailed in Fig 2.

To achieve an ideal trade-off between execution time and performance, all the models used in this work were trained without the use of any cross-validation techniques and without the use of hand-crafted feature selection techniques for machine learning models.

To evaluate the models, the metrics chosen are the standard criteria performance used in the literature: Accuracy (ACC), Sensitivity (SE), Specificity (SP), Precision (PRE), F1-score (F1), Area Under the roc Curve (AUC); all the metrics are related to the confusion matrix so to the True Positive (TP), True Negative (TN), False Positive (FP) and False Negative (FN) values:

$$\begin{array}{c} \text{ACC}=\frac{\left(TP+TN\right)}{\left(TP+FP+TN+FN\right)} \end{array}$$ (1)

$$\begin{array}{c} \text{SE}=\frac{TP}{\left(TP+FN\right)} \end{array}$$ (2)

$$\begin{array}{c} \text{SP}=\frac{TN}{\left(TN+FP\right)} \end{array}$$ (3)

$$\begin{array}{c} \text{PRE}=\frac{TP}{\left(TP+FP\right)} \end{array}$$ (4)

$$\begin{array}{c} F1=2*\frac{\left(SE*PRE\right)}{\left(S+PRE\right)} \end{array}$$ (5)

#### Decision Tree

Decision Tree (DT) is one of the most common classification methods and can be extended and used in different fields and disciplines. It can be used as a substitute for statistical processes, for machine learning, for pattern recognition, and also for various medical applications. There are two main reasons for the widespread use of DTs: *(i)* they are a very versatile type of structure because they allow a set of classification rules to be represented and *(ii)* they supports very fast computational time which depends on the depth of the tree. The structure of DT consists of nodes and edges: the root node has no incoming edges whereas internal nodes have both incoming and outgoing edges. At the last level, the nodes are called leaves and have no outgoing edges. The endpoint of each classification is the leaf node, which indicates the class assigned to the instance. Based on the value of the attribute chosen for classification, each internal node performs a test function that divides the space into two or more subspaces (subtrees). The attribute should be chosen to have a discriminating dataset partition that is useful for analysis. This process is repeated recursively on each subspace obtained until all points of the partition belong to the same class (an additional partition would not add value to the predictions). This can be checked by the use of specific heuristics, such as the Gini index, also known as Gini impurity, which measures the impurity of a node:

$$\begin{array}{c} \text{Gini}=1-\sum_{i=1}^{n}p_{i}^{2} \end{array}$$ (6)

where *p*_*i*_ is the ratio of the number of class *i* samples to the total number of training set samples. The Gini index may assume values between 0 and 1, where 0 expresses the purity of the classification (all samples belong to a single class) and 1 the random distribution of the elements. Another heuristic that can be used is entropy (theoretically, the average quality of information present in a message) which tends to 0 as the node contains instances of only one class:

$$\begin{array}{c} \text{Entropy}=-\sum_{i=1}^{n}p_{i}*{\text{log}}_{2}\left(p_{i}\right) \end{array}$$ (7)

where *p*_*i*_ is the probability of finding the element *i* in the node.

The DT was chosen as one of the most widely used machine learning approaches and in this work the Gini index performed better together with the Cart training algorithm that produces binary decision trees [12].

#### Random Forest

The Random Forest (RF) is an Ensemble Learning model in which DTs represent the main component. As the name suggests, the RF consists of a large number of DTs working as a single model. Each tree in the Random Forest makes an individual forecast and the final result is only the average of the numerical results returned by the different trees in the event of a regression problem, or the class returned by the largest number of trees if the Random Forest was used to solve a classification problem. A single tree growth algorithm introduces a random component to find the best partition not between all dataset features, but between a randomly built subset of features. The aim is to ensure the diversity of the trees. Besides, during the training, the DTs will be able to train several times on the same instances thanks to the use of the Bagging technique. On a case-by-case basis, DTs may not perform very precisely but, combined as a single model, on average the projections will be closer to the optimum.

In this paper, the RF was utilized in two different ways to analyze the results: with 60, 100, and 128 trees, and it outperformed the DT in both circumstances.

### Convolutional Neural Networks

Convolutional Neural Networks (CNNs) emerge following studies conducted between ‘58 and ‘59 by Hubel and Weisel on the visual cortex of the brain. They show that the neurons of the visual cortex have small fields of local perception that react to visual stimuli only in a limited area of the visual field, which combine to form the global visual field. The factors that contribute to the design of CNNs other than the classic Artificial Neural Networks are related to memory costs and computational times, which increase exponentially with the growth of input data. To address this issue, CNN adds two specific hidden layers: the convolutional layer and the pooling layer. The neurons present in the convolutional layer, the most important blocks of CNNs, are not fully connected to every single neuron of the previous and the next layer, thus simulating the functioning of the human brain’s visual cortex in which the neuron is only connected to the neurons of the same receptive macro-area. This layer is intended to learn and to recognize patterns with a high degree of accuracy (curves, angles, squares, etc.) through the use of specific filters. On the other hand, the pooling layers do not have weights (they cannot therefore learn), but use subsampling techniques to minimize the computation load and memory during training by reducing the size of the previous layer. The goal is also to minimize the risk of overfitting, given the significant reduction in weights. Therefore an input layer, followed by an alternating convolutions layer and pooling layers to finish with the output layer, forms a typical CNN structure. After the convolutional layers typically the activation function used is RELU which reduces the vanishing gradient problem and improves performance:

$$\begin{array}{c} \text{RELU}(X)=\text{max}\left(0,X\right) \end{array}$$ (8)

In case *X* is negative, this function will assume value 0, otherwise it will assume value *X*. This implies that only a positive input is propagated forward. For the output layer the Softmax activation function is used to classify the input data in different groups:

$$\begin{array}{c} \sigma \left(s\left(X\right)\right)=\frac{e^{s(X)}}{\Sigma e^{s(X)}} \end{array}$$ (9)

where *s*(*X*) denotes the score for instance *X*. Softmax is a normalized exponential function that returns the class with the maximum probability.

Since the 1980s, CNNs have been used for different purposes, including image detection, speech recognition, and so on, and many models have been selected for this study. The CNNs chosen in this work are trained using the transfer learning technique. This technique, which considerably reduces the computation time, allows one to reuse most of the parameters of the networks already trained previously on similar problems, concentrating on the modification and training only of the last layers. Furthermore, it favours and simplifies the experiment reproducibility.

#### ShuffleNet

ShuffleNet, introduced by Zhang *et al.* [13], is a CNN specifically designed for devices with limited computing power, as in the clinical settings considered in this study. To achieve excellent results even in presence of devices with limited computing resources, the purpose is to determine the best trade-off between speed and accuracy.

CNNs, such as ResNet, are designed through the repetition of convolutional groups that consistently increase the complexity of the network, the size, and the number of parameters used. The ShuffleNet architecture model, described in Table 1 is based on the residual unit structure by introducing two new operations: point-wise group convolution and channel shuffle to reduce complexity and time of execution.

**Table 1:** ShuffleNet architecture.

Layer	Output Size	Kernel Size	Stride	Repeat
image	224×224			
conv1	112×112	3×3	2	1
maxPool	56×56	3×3	2	
stage2	28×28		2	1
stage2	28×28		1	3
stage3	14×14		2	1
stage3	14×14		1	7
stage4	7×7		2	1
stage4	7×7		1	3
globalPool	1×1	7×7		
**fullyConnected**	2			
**softmax**	2			
**classification**	2			

**Fig 3:**
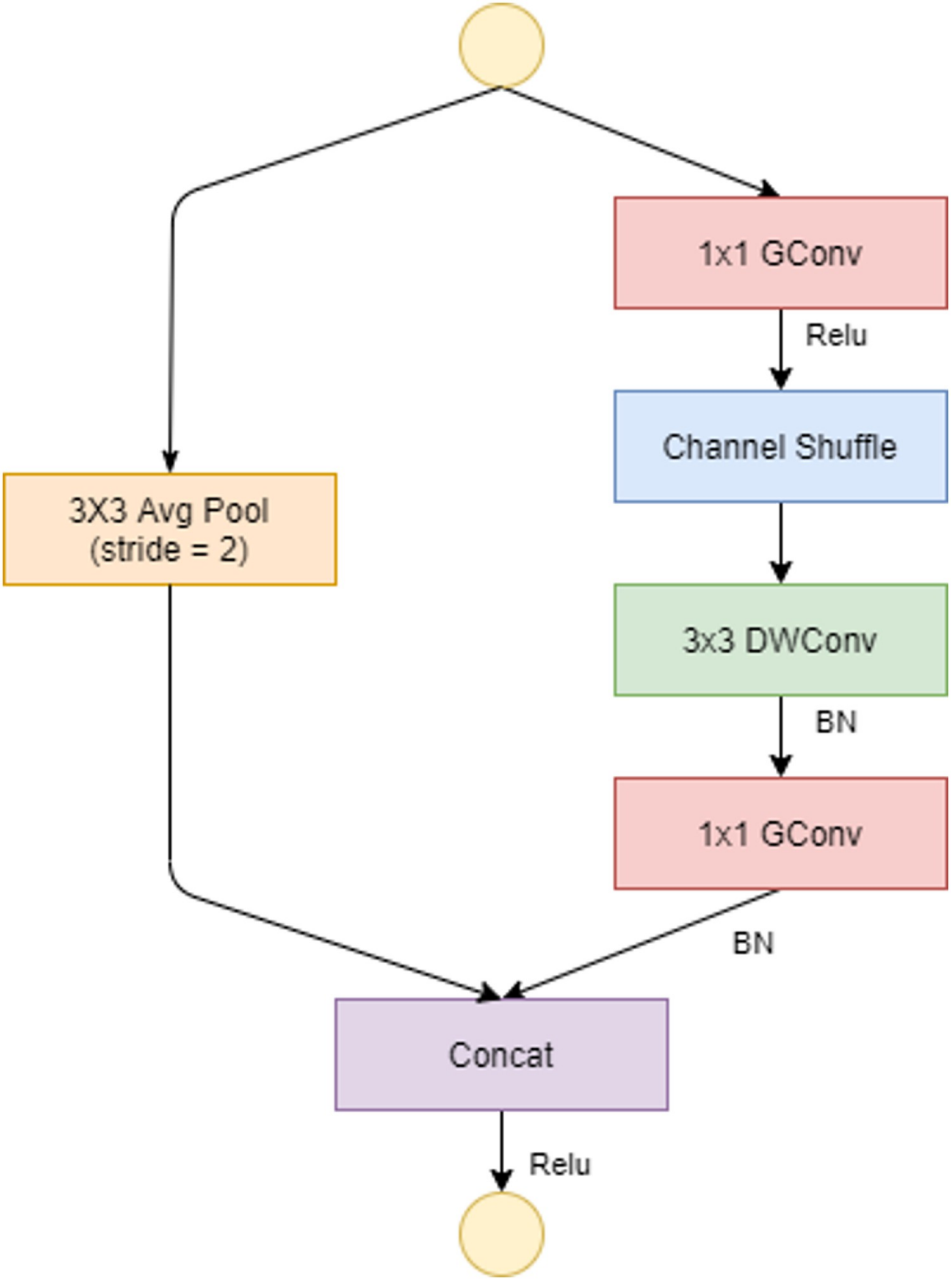
ShuffleNet unit with stride = 1. The activation functions used are Relu and Batch normalization (BN).

**Fig 4:**
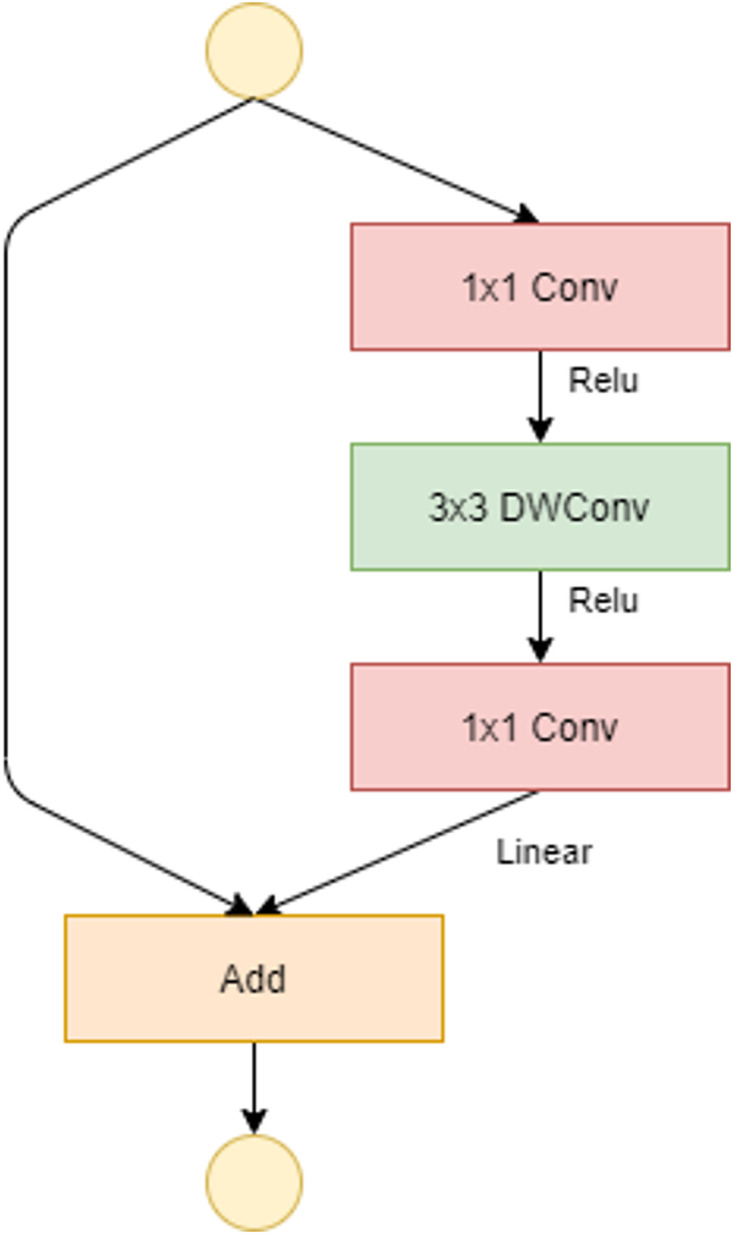
ShuffleNet unit with stride = 2.

To allow a correct correlation between input levels and output levels, in the ShuffleNet unit, the dense 1×1 convolution layer is replaced by the 1×1 point-wise group convolution (GConv) layer. The channel shuffle comes after the 1×1 GConv layer, followed by the 3×3 depthwise convolution layer (DWConv), and finally the GConv layer, which resets the channel size to match the size of the shortcut path. The DWConv layer differs from normal convolutional operations in that it aims to distinguish the depth and spatial dimensions to produce excellent results while reducing computational costs. One shuffle channel is necessary in the ShuffleNet unit because it allows for the correct exchange of information between convolution groups. If stride(the amount of movement over the image or video) = 1, as shown in Fig 3, the input and output data can be added directly; if stride = 2, the number of channels increases and the input and output data cannot be matched through the added element; however, two changes must be made: add the 3×3 AVG Pool layer on the input to the shortcut path so that the number of feature maps is the same size as the output and the last element is the concatenation channel, as defined in Fig 4.

In addition, Table 1 describes also the ShuffleNet units which are represented by three stages: *(i)* stage2, *(ii)* stage3, and *(iii)* stage 4, which differ in output size. The modified layers are highlighted in order to adapt the network to the dataset used.

In this work, ShuffleNet was chosen because its essence is to minimize convolution operations by having a model that significantly reduces complexity while maintaining the highest accuracy.

#### ResNet-18

Most of the very CNNs are affected by the convergence problem with an increase in the network’s depth. The residual block solved this problem. The design of the Residual Network (ResNet), shown in Table 2, has allowed very deep neural networks to be trained and to avoid gradient problems [14].

**Table 2:** ResNet-18 architecture.

Layer	Output Size	Kernel Size	Stride
conv1	112×112×64	7x7, 64	2
conv2_x	56×56×64	3×3 maxPool	2
		$[\begin{array}{c} 3\times 3,64\\ 3\times 3,64 \end{array}]\times 2$	
conv3_x	28×28×128	$[\begin{array}{c} 3\times 3,128\\ 3\times 3,128 \end{array}]\times 2$	
conv4_x	14×14×256	$[\begin{array}{c} 3\times 3,256\\ 3\times 3,256 \end{array}]\times 2$	
conv5_x	7×7×512	$[\begin{array}{c} 3\times 3,512\\ 3\times 3,512 \end{array}]\times 2$	
avgpool	1×1×512	7×7	
**fullyConnected**	2	512×2	
**softmax**	2		
**classification**	2		

The definition of the skip connection reflects the fundamental turning point of the residual block: the introduction of interactions between neurons belonging to non-adjacent layers. The skip connection, which is an identity mapping, has no parameters but is used to add to the last level of the jump, the output of the previous level (*F*(*x*)) and the output of the first level (*x*) from which it starts the jump. This allows the model to learn correctly and to avoid a gradient’s problem:

$$\begin{array}{c} y=F(x,W_{i})+x \end{array}$$ (10)

where *F*() represents the residual mapping. The ResNet chosen is ResNet-18, which has 18 deep layers and represents an efficient trade-off between the computation times and the final performance achieved.

#### MobileNetv2

MobileNetv2 was introduced by Sandler *et al.* [15]. It is a CNN designed specifically for devices and environments with limited computational resources. The network structure, shown in Table 3, is based on the previous version of MobileNetv1, which introduced the concept of Depthwise Separable Convolutions (DWConv), a key block for many neural networks.

**Table 3:** MobileNetv2 architecture.

Layer	Output Size	Kernel Size	Expansion Factor	Stride	Repeat
image	224×224×3			2	1
conv2d	112×112×32			2	1
bottleneck	112×112×16		1	1	1
bottleneck	56×56×24		6	2	2
bottleneck	28×28×32		6	2	3
bottleneck	14×14×64		6	2	4
bottleneck	14×14×96		6	1	3
bottleneck	7×7×160		6	2	3
bottleneck	7×7×320		6	1	1
conv2d	7×7×1280	1×1			1
avgpool	1×1×1280	7×7			1
**fullyConnected**	2				
**softmax**	2				
**classification**	2				

With DWConv, the full convolution operator is replaced by a factored version so dividing convolution into two separate layers. The first is a DWConv that performs a single filter on input data while the second, GConv, is a 1×1 convolution that builds new features through a linear combination. The aim is to significantly reduce the cost of complexity and the size of the network model. For MobileNetv2, 3×3 DWConv are used to further reduce computational costs.

**Fig 5:**
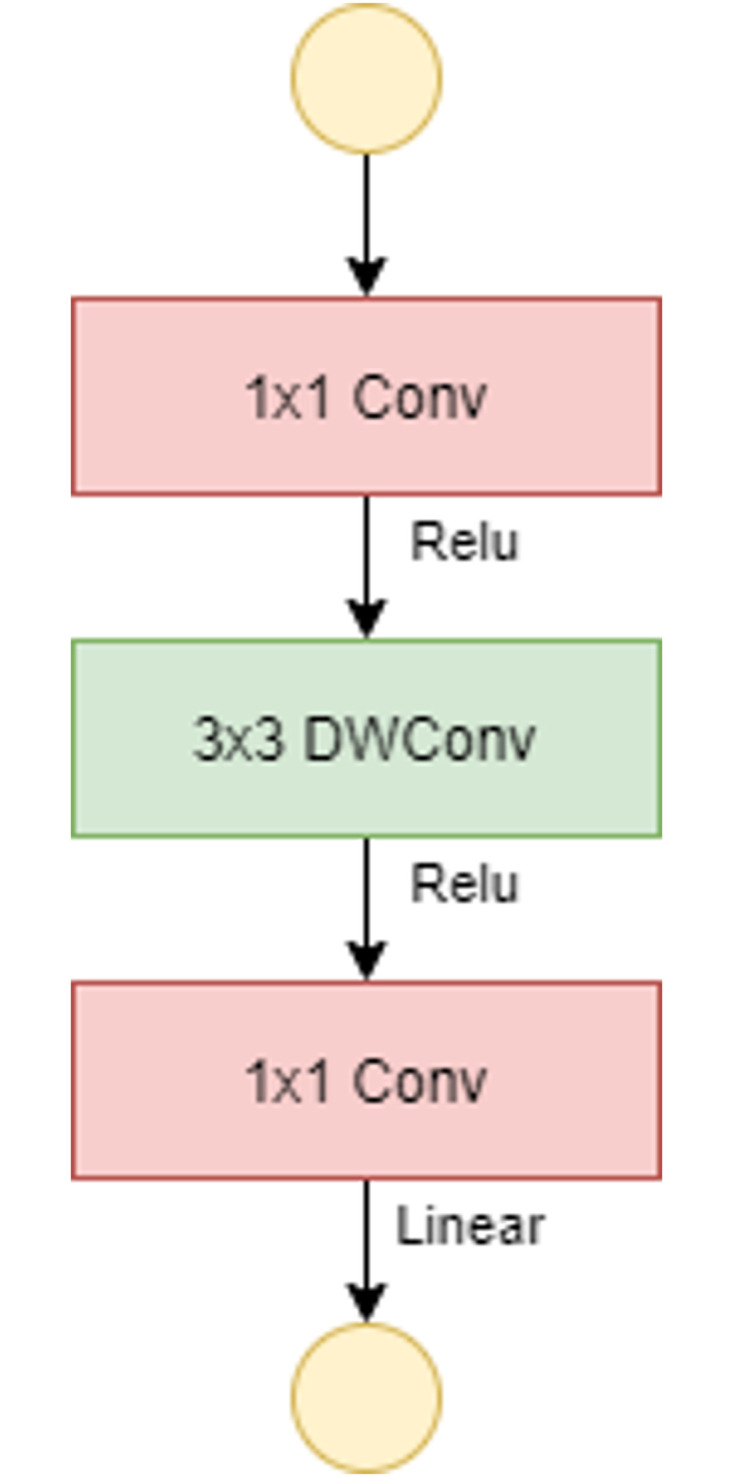
Bottleneck residual block of MobileNetv2 with stride = 1.

**Fig 6:**
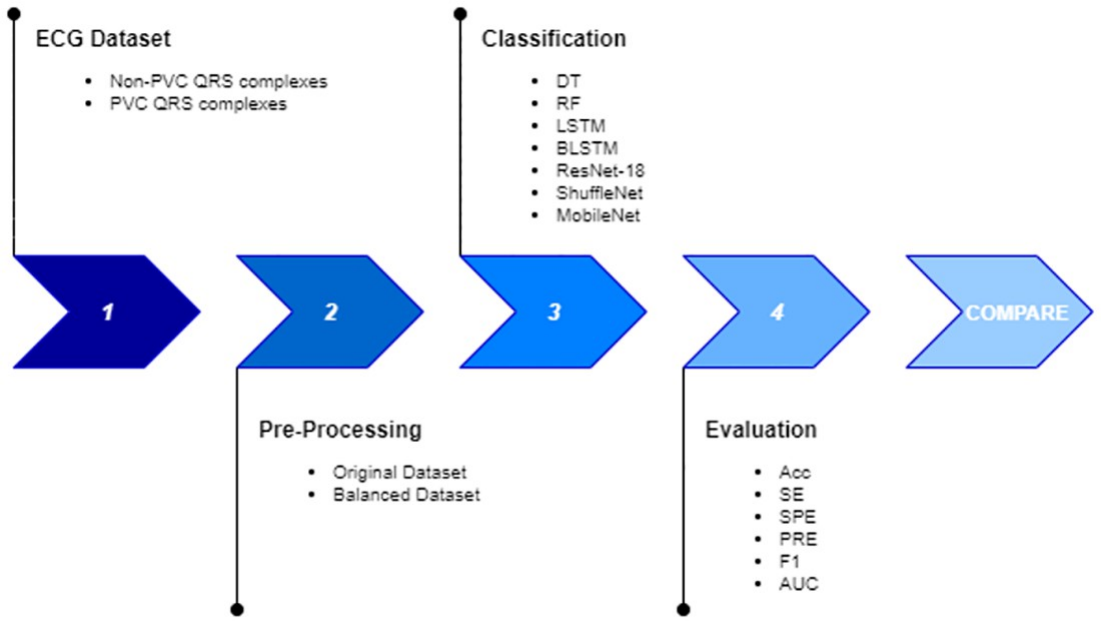
Bottleneck residual block of MobileNetv2 with stride = 2.

The introduction of a new layer module is the absolute novelty: the inverted residual with linear bottleneck. Similar to the residual block, it is based on two insights: *(i)* the input manifold can be encoded in low-dimensional subspaces, *(ii)* the non-linear activation functions cause information loss only if the input manifold does not have a low input space in the subspace. Fig 5 presents the bottleneck residual block just mentioned. This block consists of a first 1×1 conv layer (convolutional layer), followed by a 3×3 DWConv and again a 1×1 conv layer. If stride = 1, as shown in Fig 5, the input and output data can be added directly. As in the residual block standard, the addition of shortcuts represents an attempt to improve the gradient’s spread capacity. On the other hand if stride = 2, there is no shortcut path because input and output cannot be added directly (Fig 6). There are 19 bottleneck residual blocks in the final network architecture. The resulting structure, reduces the use of memory and enables very powerful inferences to be accessed. In our case, MobileNetv2 proved to be the best classifier in both experiments.

### Recurrent Neural Networks

As opposed to the feed-forward networks where the signal moves from the input level to the output level, Recurrent Neural Networks (RNNs) introduce a feedback loop that allows the newly processed output to be fed back into the network as a new input. Essentially, RNNs provide for backward links. The feedback loop allows the network to process, learn and predict sequential data that may vary in length and are not independent of each other but related to the data that precedes them. The concept of recurrence intrinsically introduces the concept of network memory: output can influence itself in a subsequent step, or it can influence the neurons of the previous chain, which in turn interferes with the neurons on which the loop closes.

The RNN replaces conventional hidden layers with recurrent layers in which it will also adds its output at time *t* − 1 as the input *u*_*t*_ at time *t*. The neural network’s current state depends on the previous state and the current input given by:

$$\begin{array}{c} x_{t}=\phi \left(W^{r}x_{t}-1+W^{i}u_{t}\right) \end{array}$$ (11)

where *x*_*t*_ is the network state at time t on the *u*_*t*_ input with *W*^*r*^ and *W*^*i*^ the weights of the recurring layer and the weights of the input layer, respectively. *ϕ*() is the activation function. For a recurrent neural network the input data are time series that can be considered in many instants of time as a sampled function.

In the case of ECGs, RNNs are a popular tool due to their memorizing skills, which allow for better identification of alterations in curves, which are the main symptom of PVCs.

#### Long Short Term Memory

Long Short Term Memory (LSTM) is a particular RNN specifically designed to avoid the long-term dependency issue and is especially suitable for data-based classification, processing, and forecasting, such as time series [16]. The structure of the LSTM is similar to the vanilla RNN in which the flow of information is processed through a memory cell. In this way, the LSTM can determine what to memorize and what to forget, i.e., it can decide the relevant details for the final classification from the less important ones. The cell consists of three gates through which the flow of information is controlled and can be read, written, or deleted by weight. Each gate produces a value of 0 to 1. No knowledge may be passed by a value of 0.

As in the RNNs, the input is the output at time *t* − 1 and the input *u*_*t*_ at time *t*. To update the memory cell status, first identify the information to be deleted and then the operation is divided into two parts to decide which information to update and which to add to the cell status. When the cell status is updated, the network output is calculated:

$$\begin{array}{c} z_{t}=o_{t}*\text{tanh}\left(x_{t}\right) \end{array}$$ (12)

where *o*_*t*_ is the output gate and *x*_*t*_ is the updated cell status.

The vanishing gradient problem is solved thanks to LSTM as information is stored by the network and long-term dependencies can be modeled.

#### Bidirectional Long Short Term Memory

Bidirectional Long Short Term Memory (BLSTM) is an enhancement to conventional LSTM where two LSTMs are trained. This will provide the network with additional background and contribute to quicker and more complete learning of the problem.

The idea of Bidirectional RNN (BRNN) is simple: it uses two RNN networks where one reads the forward sequence and the other reads the reverse sequence and can be trained using all the input data available in the past and in the future of a particular time interval [17]. The forward states do not interact with the backward states and vice versa, so it is possible to use the same algorithm which is used with simple RNN. The training process follows three steps: at first, at the forward pass, the input is processed first by the forward states (from time *t* = 1 to *t* = *T*) and then by the backward states (from time *t* = *T* to *t* = 1) and only at last by the output neurons. In the second step, the backward step, the passage from the output neurons to the backward states (from time *t* = *T* to *t* = 1) and then to the forward states (from time *t* = 1 to *t* = *T*) are carried out. Finally, the weights will be updated.

The BRNN approach has been widely used with LSTMs as past and future input information processed within a specific time interval obtain better results than linear interpretation without including excessive time delays in the processing of future information.

Table 4 summarizes the architecture of the LSTM and BLSTM developed for our purpose.

**Table 4:** LSTM and BLSTM architecture implemented in Matlab without transfer learning technique.

LSTM	Output Size	BLSTM	Output Size
SequenceInput	1	SequenceInput	1
Lstm	50	Blstm	50
**fullyConnected**	2	**fullyConnected**	2
**softmax**	2	**softmax**	2
**classification**	2	**classification**	2

### Experiments setup

All tests were performed on a Windows 10 laptop equipped with i7-8550U CPU, 16 GB RAM, and the NVIDIA MX150 GPU (2GB),. Matlab, a programming interface, has been used for machine learning algorithms. The optimization is provided by the *Adam* algorithm for LSTM and BLSTM with a learning rate of 0.005, while the optimization is given by the *SGDM* algorithm with a learning rate of 0.001 for ResNet-18, ShuffleNet, and MobileNetv2. The number of epochs and hyperparameters depend on the time and memory costs needed by the model.

## Results and discussion

This section presents and discusses the results of the various models detailed in the Method section. The number of epochs and hyperparameters can be changed depending on the computation time necessary. Three types of RF studies were carried out, one with 60 DT, one with 100 DT, and the last with 128 DT, as shown in the Tables 5 and 6. On the two types of datasets, these types of experiments were carried out in order to accurately evaluate the RF’s performance in various situations. The results for the various metrics reveal that the performances are slightly different from one another, with excellent performance (above 90%) even when no features extraction approaches are used.

**Table 5:** Random forest results (1^st^ experiment).

Random Forest	ACC	SE	SP	PRE	F1	AUC
RF–60	0.9926	0.9315	0.9987	0.9865	0.9582	0.9651
RF–100	0.9927	0.9322	0.9987	0.9865	0.9586	0.9654
RF–128	0.9925	0.9308	0.9987	0.9858	0.9575	0.9647

**Table 6:** Random forest results (2^nd^ experiment).

Random Forest	ACC	SE	SP	PRE	F1	AUC
RF–60	0.9783	0.9783	0.9987	0.9783	0.9783	0.9783
RF–100	0.9804	0.9797	0.9811	0.9811	0.9804	0.9804
RF–128	0.9804	0.9804	0.9804	0.9804	0.9804	0.9804

LSTM and BLSTM have been trained with two different values using both for 500 and 800 epochs; the results obtained are shown in Tables 7–10.

**Table 7:** LSTM results (1^st^ experiment).

LSTM	ACC	SE	SP	PRE	F1	AUC
500	0.9938	0.9562	0.9973	0.9709	0.9635	0.9767
800	0.9922	0.9527	0.9960	0.9584	0.9556	0.9744

**Table 8:** LSTM results (2^nd^ experiment).

LSTM	ACC	SE	SP	PRE	F1	AUC
500	0.9884	0.9860	0.9908	0.9709	0.9885	0.9884
800	0.9905	0.9871	0.9938	0.9935	0.9903	0.9905

**Table 9:** BLSTM results (1^st^ experiment).

BLSTM	ACC	SE	SP	PRE	F1	AUC
500	0.9941	0.9592	0.9974	0.9715	0.9653	0.9783
800	0.9922	0.9503	0.9961	0.9586	0.9544	0.9732

**Table 10:** BLSTM results (2^nd^ experiment).

BLSTM	ACC	SE	SP	PRE	F1	AUC
500	0.9846	0.9839	0.9853	0.9853	0.9846	0.9846
800	0.9870	0.9900	0.9842	0.9837	0.9868	0.9871

It’s interesting to note that in the first experiment, both LSTM and BLSTM produced better results with only 500 epochs because the entire dataset was used for training, rendering unnecessary 800 epochs. The case with 800 epochs performs better for both LSTM and BLSTM in the second experiment, where a reduced dataset was used.

The purpose of these experiments is to determine which model among those used achieved the best performance on the dataset. Therefore, the final summary values, have been reported in Tables 11 and 12 sorted by the accuracy from the best to the worst.

**Table 11:** Final results (1^st^ experiment).

Models	ACC	SE	SP	PRE	F1	AUC
MobileNetv2	0.9990	0.9930	0.9996	0.9958	0.9944	0.9963
ResNet-18	0.9984	0.9902	0.9991	0.9909	0.9905	0.9947
ShuffleNet	0.9967	0.9727	0.9990	0.9893	0.9809	0.9858
BLSTM	0.9941	0.9592	0.9974	0.9592	0.9653	0.9783
LSTM	0.9938	0.9562	0.9973	0.9709	0.9635	0.9767
Random Forest	0.9927	0.9322	0.9987	0.9865	0.9586	0.9654
Decision Tree	0.9871	0.9234	0.9934	0.9335	0.9284	0.9584

**Table 12:** Final results (2^nd^ experiment).

Models	ACC	SE	SP	PRE	F1	AUC
MobileNetv2	0.9909	0.9895	0.9923	0.9923	0.9909	0.9909
LSTM	0.9884	0.9860	0.9908	0.9909	0.9885	0.9884
ResNet-18	0.9860	0.9874	0.9846	0.9846	0.9860	0.9860
ShuffleNet	0.9853	0.9832	0.9874	0.9873	0.9852	0.9853
BLSTM	0.9846	0.9839	0.9853	0.9853	0.9846	0.9846
Random Forest	0.9804	0.9804	0.9804	0.9804	0.9804	0.9804
Decision Tree	0.9642	0.9601	0.9684	0.9682	0.9641	0.9643

The final results show that all models performed well (above 96%), but the MobileNetv2 neural network was the top model in both experiments, ranking first with the original dataset and the balanced dataset. All these results suggest that although deeper and more efficient networks, such as ResNet-18, have shown better outcomes than MobileNetv2, but in presence of smaller datasets, the deeper network is not always more efficient.

The works at the state of the art analyzed in the Introduction use always ECG recordings extracted from the MIT-BIH database, except for one work, which uses the Chinese Cardiovascular Disease Database (CCDD) in addition to MIT-BIH. Geeta and Naveen have used only 5 files for normal beats: 100, 101, 103, 105 and 220 while six files for PVC beats: 200, 203, 213, 214, 215, 221 and 223 [7]. In Xie *et al.* [8] and Zhou *et al.* [10] the dataset, consisting of 44 records, is divided into two dataset, each containing 22 records: one for training and the other for testing. Finally, all MIT-BIH recordings except 4 pacemaker patients are used in Kim *et al.* [9]. In addition, in Geeta and Naveen [7] and Xie *et al.* [8] there is a pre-processing phase for the selection features. However, the proposed approach, outperforms the aforementioned methods. Table 13 summarizes the various performances for each work.

**Table 13:** Summary of research on the classification of PVC.

Authors	ACC	SE	SP	PRE	F1	AUC
Geeta and Naveen [7]	-	0.9800	-	0.9607	-	-
Xie *et al.* [8]	0.9638	0.9788	0.9756	0.9546	-	-
Kim *et al.* [9]	0.9864	0.9840	0.9870	-	-	-
Zhou *et al.* [10]	0.9803	0.9642	0.9806	0.9340	-	-
**Proposed Method** 1^st^ experiment	**0.9990**	**0.9930**	**0.9996**	**0.9958**	**0.9944**	**0.9963**
**Proposed Method** 2^nd^ experiment	**0.9909**	**0.9895**	**0.9923**	**0.9923**	**0.9909**	**0.9909**

Even if there is no feature selection or cross-validation technique within the various recordings, our approach has the best performance. Differently from the results in [18] where DT outperformed all other models, in this work, the DT ranked the worst in both experiments, since the amount of data to be processed was too high and did not allow the model to achieve high performance. Therefore, the deeper model is not always the best performing model, as it is evident from the results; however, on the contrary, the less profound models have produced better results with smaller dataset.

Classification errors (albeit in very small percentages) are motivated by the very nature of PVCs. By definition they do not have a common pattern that allows them to be identified uniquely, but the morphology is very often very similar to other arrhythmias and in some cases, it can be almost imperceptible. For this reason, classifiers very often confuse non-PVC QRS complexes from PVC QRS complexes [19]. False predictions have caught the interest of cardiologists who are always on the lookout for systems that can accurately detect arrhythmias and heart problems. These diseases are not always characterized by specific patterns immediately recognizable by the human eye, such as PVCs. Specifically, cardiologists who manually analyze long-term ECG in normal cases have a classification rate of over 90%, while technicians and other experts have a rate of just 71% [20]. However, in rare and abnormal cases, cardiologists’ classification rate can fall as low as 72% [20].

The proposed system allows for very high performance (in the range 96.4-99.9% if we consider the accuracy) while maintaining a low percentage of classification errors, emphasizing how the model chosen can influence the final diagnosis.

## Conclusion

PVC is a very common occurrence and is represented by an extra heartbeat perceived in the chest as a skipped beat. Single beat PVC is not harmful and does not require specific treatments, but frequent PVCs and associated with other cardiomyopathies may be extremely dangerous. The development and analysis of techniques for the correct detection of PVC are therefore of vital importance.

The goal of our work is to determine under what conditions classifiers produce optimal results, particularly for biomedical data, and under what conditions detection and classification produce suboptimal performance, resulting in disastrous consequences in practice.

This approach is based on a group of six classifiers: DT, RF, LSTM, BLSTM, ResNet-19, ShuffleNet, and MobileNetv2 to detect PVCs and to understand the reason for misclassification of the models. The MobileNetv2 had the highest accuracy, with 99.90% and 99% percent in the first and second experiments, demonstrating that the final results are influenced by the size of the dataset rather than the network’s power. Furthermore, the use of the transfer learning technique to train the CNNs, combined with the thorough descriptions of the other networks, makes the experiments easily repeatable.

This study provides an automatic detection system for PVCs, which is highly desired by cardiology experts. It allows for the identification of PVCs with a precision of greater than 99% (neural network models), and these results indicate that the system can be used in clinical settings, as it maintains stability even for samples that represent rare clinical conditions or samples acquired in less-than-ideal conditions. Furthermore, the system represents a fair trade-off between accuracy and cost because it employs models with low computational power, satisfying cardiologists and experts who require a simple, and, most importantly, reliable system.

The choice of avoiding the feature extraction phase on one hand does not reduce the information in the ECG signal but on the other hand, may represent limitations in case of noise, baseline bundle branch block, aberrant conduction, paced beats. The goal for future work is to expand the dataset from 1-lead to 12-lead in order to evaluate its performance and to extend the cut-off of the PVC to better capture the morphology of the waves. Future research will also focus on validating our approach by experts, which is delayed by the current COVID-19 pandemic, on detecting other cardiac problems (e.g., left bundle branch block and right bundle arrhythmia), and identifying specific patterns that can assist systems and experts to provide an early diagnosis.
